# Menorrhagia

**Published:** 1856-12

**Authors:** Thos. A. Mitchell

**Affiliations:** De Kalb, Crawford Co., O.


### TREATMENT OF MENORRHAGIA.

EDITORS MEDICAL OBSERVER:—If you deem this prescription worthy of publication, I wish you will give it a place in the *Observer;* and I hope the profession will give it a trial in the treatment of Menorrhagia, and report the result through your Journal.

I use the officinal preparations of—

℞ Tinct. Gum Kimo, ℥ij.

" Cort. Cinnamon, ℥i.

Pulv. Sulph. Cupri., ℥i.

m. et ft. solution.

I direct the patient to take ten drops of the solution three times a day, with a little sweetened water. I increase or diminish the quantity of the sulphate of copper, according to the urgency of the symptoms, or as I find the patient will tolerate. My own success with this prescription, has been satisfactory.

DE KALB, Crawford Co., O. THOS. A. MITCHELL.

